# Correlation Between Body Plethysmography and Impulse Oscillometry Across Obstructive and Restrictive Lung Diseases: Evidence from an Adult Pilot Study Cohort

**DOI:** 10.3390/diagnostics15233055

**Published:** 2025-11-29

**Authors:** Eugenia Corina Budin, Ioan Vladimir, Ruxandra Mioara Râjnoveanu, Dragoș Huțanu, Mara Andreea Vultur, Corina Mărginean, Sofia Teodora Muntean, Edith Simona Ianoși, Gabriela Jimborean, Ovidiu Simion Cotoi

**Affiliations:** 1 Pathophysiology Department, George Emil Palade University of Medicine, Pharmacy, Science and Technology of Târgu Mureș, 540142 Târgu Mureș, Romania; corina.budin@umfst.ro (E.C.B.);; 2 Faculty of Medicine, George Emil Palade University of Medicine, Pharmacy, Science and Technology of Târgu Mureș, 540142 Târgu Mureș, Romania; student.vladiqkd@yahoo.com; 3 Palliative Medicine Department, Iuliu Hațieganu University of Medicine and Pharmacy, 400371 Cluj-Napoca, Romania; 4 Pulmonology Department, George Emil Palade University of Medicine, Pharmacy, Science and Technology of Târgu Mures, 540139 Târgu Mures, Romania; dragos.hutanu@umfst.ro (D.H.); edith.ianosi@umfst.ro (E.S.I.);; 5 Oncology and Palliative Care Department, George Emil Palade University of Medicine, Pharmacy, Science and Technology of Târgu Mureș, 540139 Târgu Mureș, Romania

**Keywords:** body plethysmography, impulse oscillometry, hyperinflation, small airway dysfunction, obstructive lung disease, restrictive lung disease

## Abstract

**Background/Objectives**: Despite the complementary insights provided by body plethysmography and impulse oscillometry, direct comparisons across obstructive and restrictive lung diseases remain limited. The aim of this study was to evaluate correlations between plethysmographic and oscillometric parameters, with a particular focus on hyperinflation and small airway dysfunction. **Methods**: We retrospectively analyzed 69 adult patients (35 obstructive, 34 restrictive) hospitalized in the Pulmonology Department of Mureș Clinical Hospital. All patients underwent body plethysmography (sRaw, Rtot, FRC, RV, TLC, RV/TLC) and impulse oscillometry (R5, R20, R5-20, X5, Fres, AX). Non-parametric tests were used to compare groups, and associations were assessed using Spearman’s correlation. **Results**: Higher airway resistance (sRaw 124 vs. 62, *p* < 0.001; R5 0.55 vs. 0.38, *p* < 0.01) and greater hyperinflation (RV 124 vs. 99, *p* < 0.001) were observed in patients with obstructed airways. Impulse oscillometry reactance markers (X5, Fres, AX) significantly differentiated obstructive from restrictive pathophysiology (*p* < 0.02). In the obstructive group, sRaw correlated with R5 (*p* = 0.01) and R5-20 (r = 0.58, *p* < 0.001), while AX correlated with RV (r = 0.59, *p* < 0.001). Restrictive patients revealed negative correlations between AX and static volumes (RV, TLC, RV/TLC; all *p* < 0.05). DLCO was higher in obstructive patients (75 vs. 62, *p* = 0.01). **Conclusions**: Our study demonstrates that body plethysmography and impulse oscillometry provide complementary information on respiratory mechanics and that the results obtained with the two methods correlate significantly, especially in obstructive diseases.

## 1. Introduction

Body plethysmography is an advanced technique for measuring static pulmonary volumes and overall airway flow resistance. It allows the assessment of residual functional capacity (FRC) and specific airway resistance (sRaw). By adding deep exhales and inspirations, we can determine total lung capacity (TLC) and residual volume (RV) [1]. The basic principle of this technique is the Boyle–Mariotte law. According to this law, under isothermic conditions, the gas volume varies inversely with an increase in pressure. Therefore, the determination of respiratory volumes is possible if the lung is considered as a closed compartment and alveolar pressure changes are measured in parallel with volume variations. Alveolar pressure can be determined by measuring the pressure generated in the oral cavity during the respiratory effort, given that the airflow is blocked by the shutter. Thus, the pressure in the oral cavity is implicitly equal to that at the alveolar level [1]. Conventionally, we can define sRaw as the variation in the pressure measured inside the cabin (ΔP cabin) relative to the variation in the flow rate measured at the oral cavity level. K_p_ is a factor corresponding to the cabin volume (Vcabin) [1,2].

Impulse oscillometry is a non-invasive method for measuring the mechanical properties of the respiratory system by applying pressure or oscillating flux signals to the oral cavity during normal breathing [3,4]. Low-amplitude oscillations are superimposed on the current volume to evaluate the pressure/flow relationship frequency dependence within the respiratory impedance (Zrs) [5,6]. The impedance reflects the totality of the forces of resistance, elasticity, and inertia that must be overcome to generate the flow through the airway. For its application in clinical practice, Zrs consists of respiratory system resistance (Rrs) and reactance (Xrs). The reactance is composed of the elasticity of the respiratory system, representing the response of the lung parenchyma to the stiffness of the tissue and chest to changes in volume [3,7,8]. Pulmonary computational models were used to confirm that the difference in resistance in the range of 5–20 Hz is the optimal physiological indicator for detecting pathology in low-caliber airways [9].

The interpretation of airway resistance should take into account that hyperinflation in COPD patients is significantly linked to a significant increase in sRaw [10]. sRaw and RV are statistically significantly correlated with the severity and extent of pulmonary emphysema [11]. Regarding asthma, plethysmography has significant sensitivity in detecting pulmonary functional changes during challenge tests with methacholine. Thus, taking into account the negative predictive value, we can exclude asthma with sufficient certainty [12,13]. Impulse oscillometry is recommended for patients who are unable to perform forced maneuvers within spirometry [1]. The area under the reactance curve (AX) and R5-20 are useful to support the diagnosis of asthma and to monitor the effectiveness of either inhaled corticosteroid (ICS) therapy in combination with long-acting beta agonists or biological therapies [7].

COPD patients have a significant increase in resistance (Rrs) and reactance (Xrs) of the respiratory system compared to the healthy population, being proportional to the degree of airway obstruction. Oscillometry can play a key role in detecting the effects of smoking early, even before a diagnosis of COPD, by determining small airway damage. While the parameters studied by means of spirometry may have normal values, using oscillometry, we can detect pathological values of R5 and X5 [7].

In restrictive ventilatory dysfunctions, most studies have focused on interstitial lung pathologies. The pattern of this type of pathology is an increase in Rrs, with an increased frequency dependence of resistance and negative Xrs at low frequencies in association with an increase in elasticity. These values correlate with the severity of the restricted character assessed with TLC or VC and the severity detected radiographically [7].

The main aim of this study was to investigate how oscillometry differs from body plethysmography in assessing airway resistance. In this regard, sRaw was determined in the case of body plethysmography and R5, R5%, R20, R20%, and Di5-20 regarding impulse oscillometry.

The secondary objectives were to investigate the differences between the sample of patients with obstructive pathologies and the sample with restrictive pathologies in terms of airway resistance and static volumetric parameters and to examine whether there are correlations between these variables. Thus, we aimed to determine correlations between AX, X5, RV, TLC, and RV%TLC_SB.

## 2. Materials and Methods

We conducted a retrospective study analyzing data from 1 January 2018 to 30 September 2024 obtained through the Hipocrate H3 concept-integrated computer system at the Pneumology Department of Mures County Clinical Hospital. This study received approval from the Ethics Committee (approval no. 15359/28 October 2024). The information collected was as follows: gender; age; body mass index (BMI); main diagnosis and secondary diagnoses; exposure to pollutants; body plethysmographic parameters, including sRaw, RV, TLC, and RV%TLC_SB; and impulse oscillometry parameters, including R5, R5%, R20, R20%, R5-20, AX, and X5.

The inclusion criteria were adult patients over the age of 18 who were hospitalized in the Pneumology Department with a confirmed diagnosis of asthma, COPD, or diffuse interstitial lung disease (ILD), and who had given their consent to performing exploratory maneuvers. Every patient diagnosed with asthma in their medical history had a spirometry with a bronchodilator test.

We excluded patients who did not consent to the exploratory maneuvers, those who did not have body plethysmography or impulse oscillometry performed, and those with contraindications, namely, severe respiratory failure, hemoptysis, pneumothorax, uncontrolled cardiovascular disease (arterial hypertension, acute coronary syndrome), aneurysm, facial trauma, retinal detachment, glaucoma, or claustrophobia. Moreover, patients with a mixed pattern on functional samples (both obstructive and restrictive) were excluded to avoid confusion in the statistical analysis.

We analyzed a database of 106 patients in the Pneumology Department of Mures Clinical County Hospital during the study period. Following the inclusion and exclusion criteria, the final study sample included 69 patients. Subsequently, the sample was divided into two analysis groups: 35 patients with obstructive pathology and 34 patients with restrictive pathology. This study was carried out in accordance with the confidentiality provisions regarding the processing of personal data and with the principles stated in the Declaration of Helsinki.

Statistical data analysis was performed using IBM SPSS Statistics, version 26.0.0.0. The distribution of data was verified based on the evaluation of kurtosis and skewness parameters and inspection of histograms and Q-Q plots and finally corroborated by the Shapiro–Wilk distribution test. All analyses confirmed a non-parametric distribution of data. Therefore, the quantitative data are expressed in the form of the median, along with the minimum/maximum interval (min–max), while the qualitative data are expressed in the form of absolute values, along with the relative value for *n* (%).

For the analysis of statistical differences, the Mann–Whitney U and Kruskal–Wallis tests were performed as appropriate for quantitative data; for qualitative data, the chi-square test was used. The presence of correlations between the studied variables was tested using the Spearman correlation coefficient, with 0 ≤ r < 0.3—weak correlation, 0.3 ≤ r < 0.7—moderate correlation, and 0.7 ≤ r ≤ 1—strong correlation as the reference values. The statistical significance threshold was set at α = 0.05. For multiple comparisons in Table 1, we applied the Bonferroni correction to control the familywise type I error rate. With 22 independent tests, the corrected significance threshold was α = 0.0023.

## 3. Results

This study included 69 patients, with a median age of 59 (51–70) years and a predominance of the female gender (*n* = 41 (59.4%) female versus *n* = 28 (40.6%) male). Regarding patient characteristics, *n* = 32 (46.4%) had a history of occupational exposure to respiratory noxious gases, and *n* = 35 (50.7%) had obstructive syndrome, of whom *n* = 8 (11.59%) had a diagnosis of COPD and *n* = 27 (39.13%) had a diagnosis of asthma (confirmed with a spirometry test with a bronchodilator response), and 34 (49.3%) had a diagnosis of restrictive syndrome. Regarding complex specific treatments, *n* = 11 (15.9%) patients received antifibrotic treatment, and *n* = 6 (8.7%) patients received treatment with biological monoclonal antibodies for asthma. The plethysmographic evaluation revealed significant differences between patients with obstructive and those with restrictive respiratory disease. Individuals with an obstructive pattern demonstrated markedly elevated airway resistance, with both sRaw (124 vs. 62, *p* < 0.001) and Rtot (144 vs. 109, *p* < 0.01) being significantly higher compared to those with restrictive physiology. Furthermore, parameters reflecting lung hyperinflation and air trapping were consistently greater in the obstructive group, including functional residual capacity (FRC: 120 vs. 93, *p* < 0.01), residual volume (RV: 124 vs. 99, *p* < 0.001), and total lung capacity (TLC: 110 vs. 90, *p* < 0.01).

In contrast, spirometric indices embedded within plethysmography (FEV1, FEV1/FVC, and MEF50) showed higher values in restrictive patients. Specifically, FEV1 (92% vs. 75%, *p* = 0.04), Tiffeneau index (FEV1/FVC: 82.5% vs. 71.9%, *p* < 0.01), and mid-expiratory flow at 50% of vital capacity (MEF50: 91 vs. 46, *p* < 0.001) were significantly greater in the restrictive group.

Diffusion capacity was also discriminative between groups. DLCO was higher in obstructive patients (75 vs. 62, *p* = 0.01), likely reflecting the inclusion of asthmatic patients with preserved or enhanced diffusion, whereas restrictive patients, particularly those with fibrosis, exhibited impaired alveolocapillary gas transfer. Conversely, the RV/TLC single-breath ratio (RV%TLC_SB) was significantly higher in the obstructive group (107 vs. 88, *p* < 0.001), reinforcing the presence of air trapping in this cohort.

Impulse oscillometry (IOS) further emphasized these differences. Obstructive patients had significantly greater resistances at 5 Hz (R5: 0.55 vs. 0.38, *p* < 0.01) and 20 Hz (R20: 0.33 vs. 0.29, *p* = 0.01), with the corresponding percentage predicted values also elevated. The difference between low- and high-frequency resistance (ΔR5-20: 0.14 vs. 0.08, *p* = 0.01) was greater in obstructive disease, indicating frequency dependence of resistance consistent with small airway involvement. Furthermore, markers of reactance, including X5 (more negative in obstructive: –0.17 vs. –0.12, *p* = 0.01), resonant frequency (Fres: 24 vs. 18, *p* = 0.02), and area of reactance (AX: 1.79 vs. 0.74, *p* < 0.01), clearly differentiated the two groups (Table 1).

In the obstructive subgroup, stronger mechanical abnormalities were consistently linked across methods. AX correlated positively with RV (r = 0.585, *p* < 0.001), indicating that greater reactance burden was associated with more air trapping (Table 2).

At the same time, plethysmographic airway resistance (sRAW) correlated with IOS resistance markers, including R5, R5%, R20%, and R5-20, confirming that IOS sensitively captured both central and frequency-dependent resistive loads. These associations reinforce the pathophysiological link between increased reactance, air trapping, and peripheral airway dysfunction in obstructive cases (Table 2 and Table 3).

Restrictive patients displayed an opposite pattern, where AX correlated negatively with RV, TLC, and RV/TLC (all *p* < 0.05). This means that the greater the lung volumes reduced by fibrosis, the higher the reactance abnormalities became, reflecting increased parenchymal stiffness and impaired compliance rather than resistive loading (Table 4a). Unlike in obstruction, no significant correlations were seen between sRAW and IOS resistive indices, suggesting that restriction is driven primarily by reduced volumes and altered elastic recoil rather than airway resistance (Table 4b).

**Figure 1:**
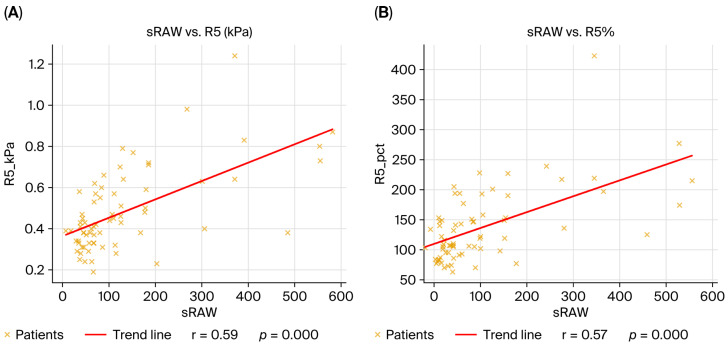

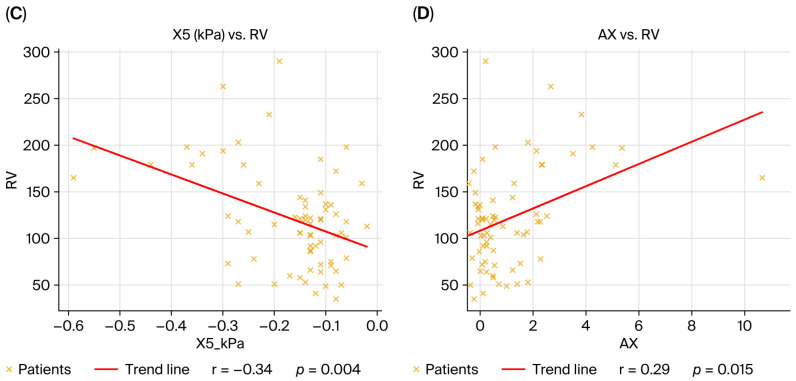
Correlations between plethysmographic and oscillometric parameters in the overall cohort (*n* = 69). (**A**) sRaw vs. R5 (kPa), r = 0.59, *p* < 0.001; (**B**) sRaw vs. R5 (% predicted), r = 0.57, *p* < 0.001; (**C**) X5 (kPa) vs. RV (% predicted), r = –0.34, *p* = 0.004; (**D**) AX vs. RV (% predicted), r = 0.29, *p* = 0.01. The linear regression fit is indicated by the red line, and each dot represents one patient. Spearman’s rank correlation coefficient was used to calculate correlations.

When all patients were analyzed together, IOS and plethysmography maintained complementary associations. More negative X5 and higher AX correlated with higher RV, linking worsening reactance to gas trapping. sRaw correlated positively with R5, R5%, R20, R20%, and R5-20, underscoring the concordance of plethysmographic resistance with IOS-derived resistances. These cross-phenotype correlations highlight the ability of IOS to reflect both resistive and reactive abnormalities that parallel static volume changes captured with plethysmography (Figure 1).

**Figure 2:**
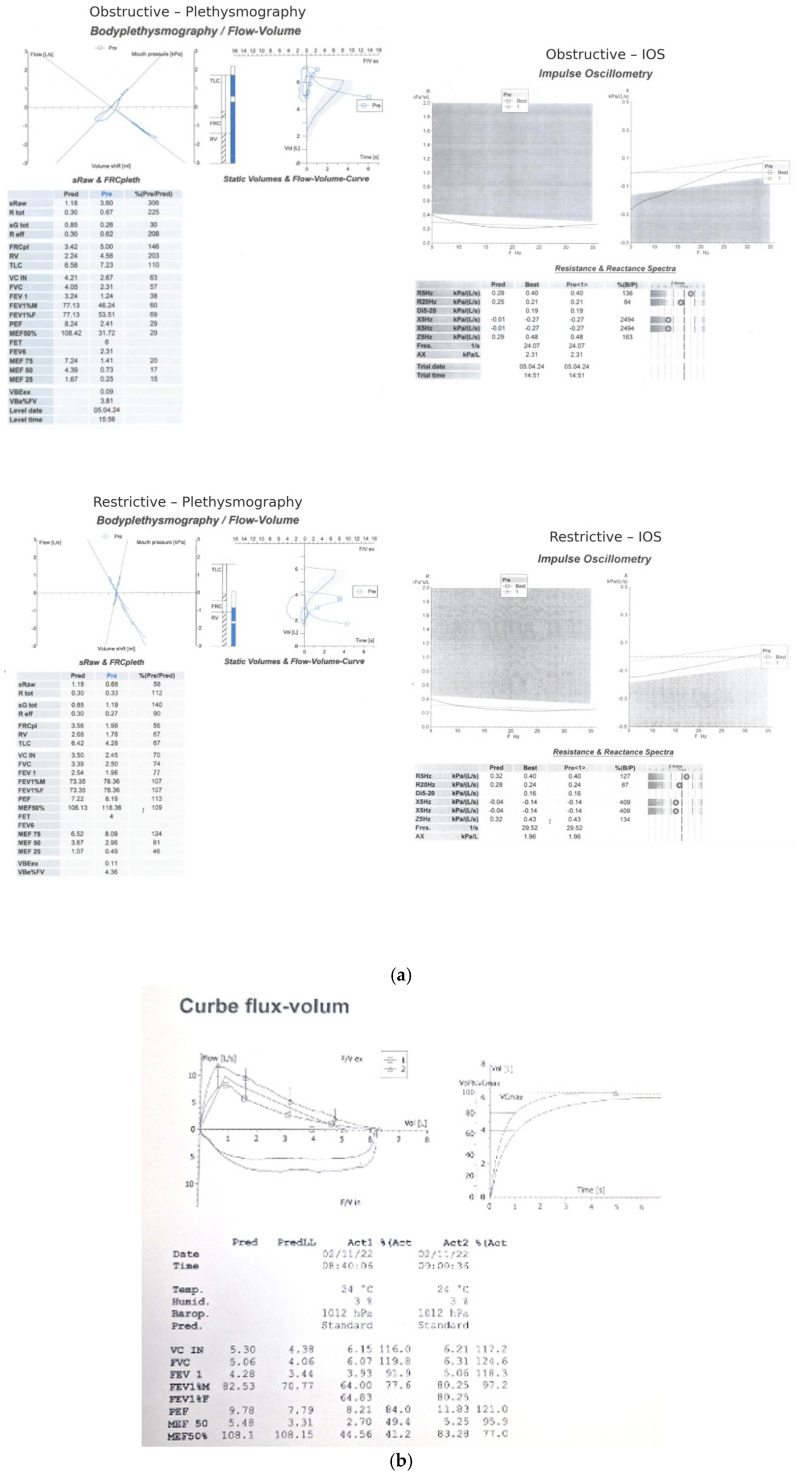
(**a**) Representative tracings of body plethysmography and impulse oscillometry from an obstructive patient (A,B) and a restrictive patient. The obstructive case demonstrates increased airway resistance (sRaw, Rtot), hyperinflation (RV, RV/TLC), and marked frequency-dependent resistance (ΔR5-20) with negative reactance (X5) and elevated AX. The restrictive case shows reduced static lung volumes (TLC, RV), preserved or mildly elevated resistance, and reactance changes consistent with reduced parenchymal compliance. (**b**) Representative pre-post spirometry.

To provide a more detailed illustration of the physiological patterns mentioned previously, Figure 2 presents the plethysmographic and impulse oscillometry tracings of an obstructive and a restrictive patient.

**Figure 3:**
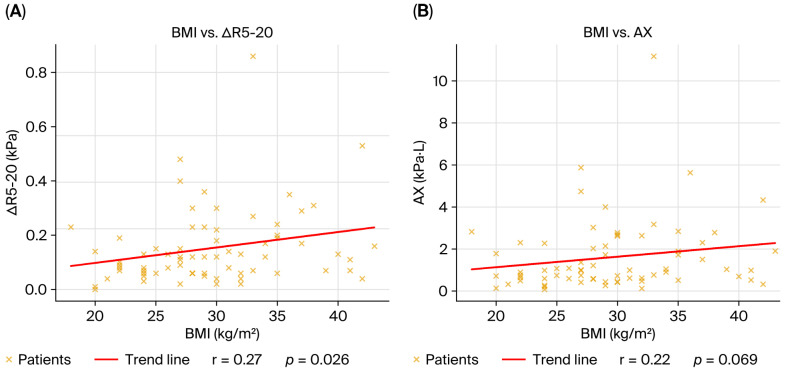
Correlations between BMI and oscillometric parameters in the overall cohort (*n* = 69). (**A**) BMI vs. R5-20, positive correlation (ρ = 0.27, *p* = 0.02); (**B**) BMI vs. AX, positive correlation (ρ = 0.22, *p* = 0.04). Each dot represents one patient; the red line indicates the linear regression fit. Correlations were calculated using Spearman’s rank correlation coefficient.

Body mass index correlated positively with R5-20 and AX, indicating that a higher BMI was associated with greater frequency dependence of resistance and higher reactance burden, consistent with obesity-related small airway closure and chest wall loading effects. The negative but nonsignificant correlations with RV and RV/TLC suggest that BMI did not contribute strongly to classical air trapping in this population (Figure 3).

**Figure 4:**
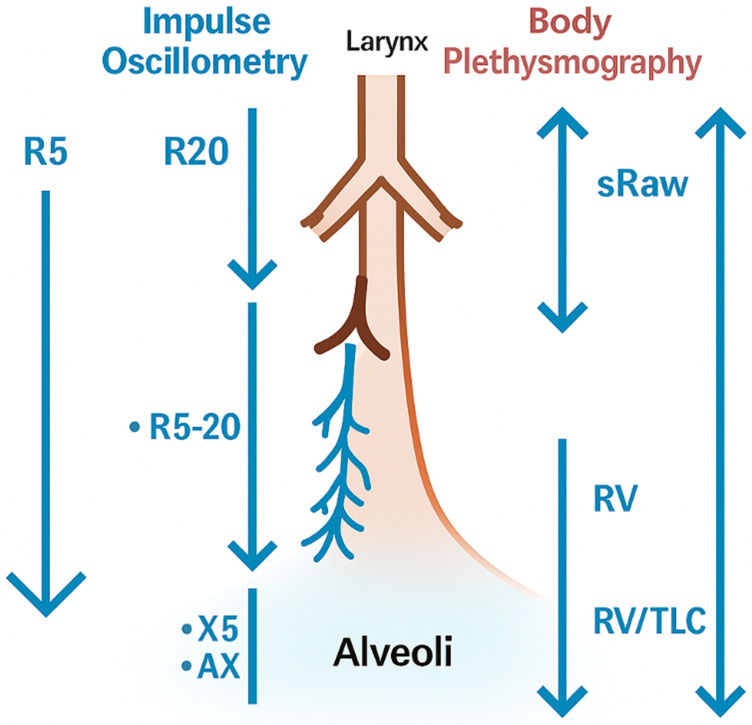
Schematic representation of IOS and plethysmographic parameters. The schematic illustrates how specific IOS indices (R20, R5, ΔR5-20, X5, AX) and plethysmographic measures (sRaw, RV, RV/TLC, TLC) relate to different levels of the airway tree, from central to peripheral airways and alveoli. The figure highlights the complementary physiological information provided by both techniques.

Figure 4 integrates IOS and plethysmographic parameters into an anatomical framework to help interpret these findings, depicting the airway locations to which every index is most physiologically linked. These anatomical considerations help contextualize the correlations described above and provide a basis for the physiological interpretation mentioned in the Section 4.

## 4. Discussion

Previous studies have explored the relationship between spirometry, body plethysmography, and impulse oscillometry (IOS), showing that IOS can differentiate asthmatic patients from healthy controls [7] and may be particularly useful in detecting distal airway dysfunction even in the presence of normal spirometry [14,15,16]. In pediatrics, direct comparisons between plethysmography and IOS have demonstrated consistent correlations between measured variables [17] while adult studies confirmed significant associations of resistance measures across both techniques [18,19,20]. Furthermore, both approaches have been demonstrated to show physiological changes after bronchodilator therapy in COPD and asthma and are considered complementary tools for assessing airway mechanics [20,21].

In our study, we confirmed significant correlations between plethysmographic and oscillometric parameters, particularly in obstructive lung diseases. Specific airway resistance (sRaw) correlated strongly with R5 and R5% predicted, reflecting global airway resistance, while its association with R20 and R20% was weaker, consistent with the fact that these parameters primarily represent central airway resistance. In contrast, no significant correlations were identified between TLC or RV/TLC_SB and oscillometry parameters, possibly due to overstatement of TLC by plethysmography, especially in patients with severe obstruction [22], or the variation in pressures measured with the mouthpiece. However, the literature reports a moderate correlation between AX and RV/TLC_SB [23]. Unexpectedly, X5 did not show a strong correlation with RV or RV/TLC in our cohort. This may be explained by the heterogeneity of the obstructive pathologies examined, variability of Xrs during tidal breathing, and the presence of mixed obstructive/restrictive patterns. Although data from the literature support moderate correlations between X5 and static lung volumes [7], our findings underline the need for further investigation with larger, more homogeneous samples.

We also observed significant relationships between lung volumes and oscillometric indices of reactance. Residual volume (RV) correlated positively with AX, highlighting the contribution of hyperinflation and premature airway closure to small airway dysfunction. Interestingly, in the restrictive cohort, AX correlated negatively with RV, TLC, and RV/TLC_SB, consistent with reduced compliance and increased stiffness associated with fibrotic interstitial lung disease. These findings are in line with previous reports showing that IOS parameters, particularly AX and X5, may reflect the mechanical consequences of fibrosis [10].

Our results confirm that both body plethysmography and impulse oscillometry correlate statistically significantly in the obstructive group, which was expected, as both methods are used to investigate respiratory mechanical properties and reflect the same pathophysiological changes. Elastic recoil loss from COPD—which causes airway narrowing during exhalation [24], early airway closure, and mechanical compression [10]—can be assessed using both plethysmographic (sRaw) and oscillometric parameters (R5, R5%, R20, R5-20). In our analysis, sRaw positively and significantly correlated with R5 and R5%, supporting the fact that these parameters reflect the total strength of the bronchial tree. Lower correlations with R20 and R20% are explainable, since they mainly describe the strength of large airways.

The strong correlation between the difference in R5-20 and sRaw (*p* < 0.001), comparable to that between sRaw and R5%, is an important finding. Although it seems paradoxical that a derived index of peripheral resistance correlates more closely with total resistance than with central pathway parameters, this observation can be explained by the derived nature of R5-20 and the heterogeneity of airway damage. Both methods appear to be sensitive to diffuse changes in small airways.

In the restrictive group, AX correlated negatively and significantly with RV, TLC, and RV/TLC_SB, reflecting the increase in pulmonary stiffness associated with fibrosing interstitial diseases, consistent with data in the literature [25,26]. Conversely, the correlations between X5 and static volumes were weak and insignificant, which can be explained by mild restrictive forms or by overlapping an obstructive component. Moreover, the correlations between sRaw and IOS parameters were weak and insignificant, a predictable aspect as restrictive diseases are characterized mainly by decreased volumes and compliance, not by increased resistance. Subgroup analysis showed treatment-related differences (antifibrotic, extrafine inhalers), but the small sample size did not allow definite conclusions to be made. In the recent literature, it has been reported that in the evolution of respiratory diseases, IOS parameters may change earlier than those of plethysmographics, suggesting an increased sensitivity of oscillometry to small airway alterations [27]. This opens new perspectives for the use of IOS as a complementary tool, both in obstructive and restrictive diseases. Despite its importance in quantifying lung volumes, hyperinflation, and airway resistance, comparative studies indicate that whole-body plethysmography is not as effective in detecting early small airway dysfunction. In contrast, IOS is consistently linked more closely to CT-based metrics of peripheral airway disease. Park et al. demonstrated significant correlations between IOS indices (R5-R20, X5, AX) and CT parametric response map abnormalities in COPD, suggesting better detection of SAD compared with spirometry or plethysmography. Huang et al. reported that IOS can detect peripheral airway impairment even when CT changes are subtle [28,29].

An additional novel finding of our study was the association between BMI and IOS markers of peripheral resistance (R5-20, AX). According to these findings, anthropometric factors may have an effect on airway mechanics, possibly through altered chest wall compliance or an increased tendency to close the airway. Moreover, subgroup analyses suggested differences related to treatment (antifibrotic therapy, extrafine inhaled formulations), although our sample size was too small to draw definitive conclusions.

A limitation of our study is the relatively small sample size, which may diminish the statistical strength and generalizability of the results. Although underpowered, subgroup analyses of asthmatic patients with biological treatment versus standard treatment and occupational exposure versus non-exposure were conducted and presented for completeness and may provide direction for future research. Moreover, the presence of extreme values can influence variability and reduce the ability to detect significant differences.

## 5. Conclusions

Our study demonstrates that body plethysmography and impulse oscillometry provide complementary information on respiratory mechanics and that the results obtained with the two methods correlate significantly, especially in obstructive diseases. Overall strength parameters (sRaw, Rtot) were found to be consistently associated with IOS markers of small airway dysfunction (R5, AX, R5-20), while hyperinflation volumes (RV, RV/TLC) showed moderate correlations with reactance parameters (X5, AX). In restrictive diseases, AX negatively correlated with static volumes, reflecting the pulmonary rigidity characteristic of fibrosis. The body mass index was also associated with the IOS parameters of peripheral resistance, suggesting an additional influence of anthropometric factors.

While spirometry has long been the cornerstone of pulmonary function assessment, modern practice requires looking beyond it. Integrating these results represents an important step toward refining clinical guidelines and advancing personalized respiratory care.

## Figures and Tables

**Table 1:** Comparative analysis between obstructive and restrictive patients.

	Obstructive Syndrome (*n* = 35)	Restrictive Syndrome (*n* = 34)	*p*
Body plethysmography			
sRaw	124 (68–301)	62 (42–88)	<0.001 (0.0004)
Rtot	144 (100–255)	109 (66–130)	<0.01 (0.0021)
FRC	120 (84–143)	93 (62–117)	<0.01 (0.0081)
RV	124 (107–179)	99 (65–122)	<0.001 (0.0009)
TLC	110 (89–118)	90 (72–106)	<0.01 (0.0064)
FVC	87 (69–108)	93 (71–108)	0.75
FEV1	75 (55–100)	92 (73–107)	0.04
FEV1/FVC	71.86 (61.36–82.37)	82.49 (79.22–86.22)	<0.01 (0.0042)
MEF50	46 (25–93)	91 (69–107)	<0.001 (0.0002)
DLCO	75 (64–87)	62 (48–78)	0.01
KCO	93 (77–106)	90 (75–101)	0.46
VA	79 (69–97)	74 (59–87)	0.06
RV%TLC_SB	107 (97–125)	88 (77–102)	<0.001 (0.0003)
Impulse oscillometry			
R5 (kPa)	0.55 (0.38–0.71)	0.38 (0.32–0.45)	<0.01 (0.0018)
R5%	147 (103–205)	107 (86–141)	<0.01 (0.0016)
R20 (kPa)	0.33 (0.28–0.42)	0.29 (0.25–0.33)	0.01
R20%	110 (90–147)	92 (82–113)	0.01
Di5-20	0.14 (0.07–0.27)	0.08 (0.06–0.14)	0.01
X5 (kPa)	−0.17 (−0.29–−0.09)	−0.12 (−0.14–−0.10)	0.01
Z5 (kPa)	0.57 (0.40–0.76)	0.41 (0.33–0.49)	<0.001 (0.0009)
Fres	24 (17–29)	18 (16–22)	0.02
AX	1.79 (0.70–2.85)	0.74 (0.52–1.29)	<0.01 (0.0012)

Bonferroni correction was applied for these comparisons.

**Table 2:** Correlation of hyperinflation parameters with Ax and X5, respectively, in the obstructive patient group.

Obstructive Syndrome	X5 (kPa)	AX
RV	r = −0.305, *p* = 0.07	r = 0.585, *p* < 0.001
TLC	r = 0.118, *p* = 0.498	r = 0.144, *p* = 0.408
RV%TLC_SB	r = −0.188, *p* = 0.28	r = 0.297, *p* = 0.08

r—Spearman’s coefficient.

**Table 3:** The correlation of sRaw with oscillometric parameters in the obstructive patient group.

Obstructive Syndrome	R5 (kPa)	R5%	R20 (kPa)	R20%	Di5-20
sRaw	r = 0.424, *p* = 0.01	r = 0.687, *p* < 0.001	r = 0.265, *p* = 0.12	r = 0.336, *p* = 0.04	r = 0.584, *p* < 0.001

r—Spearman’s coefficient.

**Table 4:** (**a**) Correlations between hyperinflation parameters and AX and X5 in the restrictive patient group; (**b**) correlations between sRaw and impulse oscillometry parameters in the restrictive patient group.

(**a**)					
**Restrictive Syndrome**		**X5 (kPa)**		**AX**	
RV		r = 0.064, *p* = 0.71		r = −0.393, *p* = 0.02	
TLC		r = 0.063, *p* = 0.72		r = −0.398, *p* = 0.02	
RV%TLC_SB		r = 0.031, *p* = 0.86		r = −0.335, *p* = 0.04	
(**b**)					
**Restrictive Syndrome**	**R5 (kPa)**	**R5%**	**R20 (kPa)**	**R20%**	**Di5-20**
sRaw	r = 0.134, *p* = 0.45	r = 0.066, *p* = 0.71	r = 0.181, *p* = 0.30	r = −0.037, *p* = 0.83	r = 0.163, *p* = 0.35

r—Spearman’s coefficient.

## Data Availability

Data are available from the corresponding author upon request due to ethical reasons and the presence of sensitive personal data in the raw database file.

## Abbreviations

The following abbreviations are used in this manuscript:

AX	Area Under Reactance Curve
BMI	Body Mass Index
BP	Body Plethysmography
DLCO	Diffusing Capacity of the Lungs for Carbon Monoxide
FEV1	Forced Expiratory Volume in 1 s
FRC	Functional Residual Capacity
Fres	Resonant Frequency
IOS	Impulse Oscillometry
MEF50	Maximal Expiratory Flow at 50% of FVC
RV	Residual Volume
RV/TLC	Residual Volume/Total Lung Capacity ratio
sRaw	Specific Airway Resistance
TLC	Total Lung Capacity
X5	Reactance at 5 Hz
Z5	Impedance at 5 Hz
R5-20	Difference between resistance at 5 Hz and 20 Hz
